# Association between enlarged perivascular spaces and cerebrospinal fluid aquaporin-4 and tau levels: report from a memory clinic

**DOI:** 10.3389/fnagi.2023.1191714

**Published:** 2023-07-20

**Authors:** Luca Sacchi, Marina Arcaro, Tiziana Carandini, Anna Margherita Pietroboni, Giorgio Giulio Fumagalli, Chiara Fenoglio, Maria Serpente, Federica Sorrentino, Caterina Visconte, Manuela Pintus, Giorgio Conte, Valeria Elisa Contarino, Elio Scarpini, Fabio Triulzi, Daniela Galimberti, Andrea Arighi

**Affiliations:** ^1^Department of Biomedical, Surgical and Dental Sciences, University of Milan, Milan, Italy; ^2^Neurodegenerative Diseases Unit, Fondazione IRCCS Ca’ Granda Ospedale Maggiore Policlinico, Milan, Italy; ^3^Center for Mind/Brain Sciences (CIMeC), University of Trento, Rovereto, Italy; ^4^Department of Medical Sciences and Public Health, University of Cagliari, Cagliari, Italy; ^5^Department of Pathophysiology and Transplantation, University of Milan, Milan, Italy; ^6^Neuroradiology Unit, Fondazione IRCCS Ca’ Granda Ospedale Maggiore Policlinico, Milan, Italy

**Keywords:** glymphatic system, aquaporin-4, cerebrospinal fluid, Alzheimer’s disease, brain perivascular spaces

## Abstract

**Background:**

Perivascular spaces (PVS) are fluid-filled compartments that dilate in response to many different conditions. A high burden of enlarged PVS (EPVS) in the centrum semiovale (CSO) has been linked to neurodegeneration. Moreover, an increase in cerebrospinal fluid (CSF) levels of aquaporin-4 (AQP4), a water channel expressed on PVS-bounding astrocytes, has been described in patients with neurodegenerative dementia. Our aim was to investigate the relationship between neurodegenerative diseases and two putative glymphatic system biomarkers: AQP4 and EPVS.

**Methods:**

We included 70 individuals, 54 patients with neurodegenerative diseases and 16 subjects with non-degenerative conditions. EPVS were visually quantified on MRI-scans applying Paradise’s scale. All subjects underwent lumbar puncture for the measurement of AQP4 levels in the cerebrospinal fluid (CSF). CSF levels of amyloid-β-1-42, phosphorylated and total tau (tTau) were also measured. Linear regression analyses were adjusted for age, sex, education and disease duration, after excluding outliers.

**Results:**

Cerebrospinal fluid (CSF)-AQP4 levels were independent predictors of total (β = 0.28, standard error [SE] = 0.08, *p* = 0.001), basal ganglia (β = 0.20, SE = 0.08, *p* = 0.009) and centrum semiovale EPVS (β = 0.37, SE = 0.12, *p* = 0.003). tTau levels predicted CSO-EPVS (β = 0.30, SE = 0.15, *p* = 0.046). Moreover, increased levels of AQP4 were strongly associated with higher levels of tTau in the CSF (β = 0.35, SE = 0.13, *p* = 0.008).

**Conclusion:**

We provide evidence that CSO-EPVS and CSF-AQP4 might be clinically meaningful biomarkers of glymphatic dysfunction and associated neurodegeneration.

## Introduction

Perivascular spaces (PVS) are fluid-filled compartments that run parallel to penetrating arterioles in the brain and lie between endothelial cells and astrocytes (Albargothy et al., 2018; MacGregor Sharp et al., 2019). PVS have been suggested as fundamental elements of the recently described glymphatic system (GS), through which the brain is cleared of interstitial fluid and waste products (Iliff et al., 2012; Rasmussen et al., 2018). They allow the flow of cerebrospinal fluid (CSF) from the subarachnoid to the interstitial space. This process is facilitated by aquaporin-4 (AQP4), a water channel densely expressed on the astrocytic endfeet processes bounding PVS (Iliff et al., 2012). PVS can dilate and become visible on magnetic resonance imaging (MRI) (Groeschel et al., 2006) in many clinical conditions, possibly because of glymphatic dysfunction (Wang et al., 2021), with some regional and sex specificity (Zhang et al., 2014; Evans et al., 2023). Enlarged PVS (EPVS) in the basal ganglia (BG-EPVS) have been primarily associated with aging and hypertension (Bown et al., 2022; Evans et al., 2023), while the burden of EPVS in the centrum semiovale (CSO-EPVS) has been linked to cerebral amyloid angiopathy pathologic changes (Perosa et al., 2022), amyloid-beta (Aβ) (Kim et al., 2021) and tau deposition (Vilor-Tejedor et al., 2021; Wang et al., 2021) and increased risk of incident cognitive decline (Paradise et al., 2021) at least partly independent from small-vessels disease (Paradise et al., 2021; Romero et al., 2022) or Alzheimer’s disease (Jeong et al., 2022).

Recently, we found increased levels of AQP4 in the CSF of patients affected by neurodegenerative dementia, which closely mirrored CSF tau levels (Arighi et al., 2022).

The main purpose of this pilot study was to retrospectively investigate the association between two putative GS biomarkers−AQP4 and EPVS−and established CSF biomarkers of neurodegeneration in a population of people with suspected dementia.

## Methods

For this study, we retrospectively recruited 70 subjects who underwent neurological workup at the Neurodegenerative Diseases Unit of the Fondazione IRCCS Ca’ Granda Ospedale Maggiore Policlinico in Milan (Italy) in suspicion of dementia.

Of them, 54 were finally diagnosed with neurodegenerative diseases−of which 39 with Alzheimer’s disease (AD), 9 with fronto-temporal dementia (FTD), 3 with Lewy-body dementia (LBD), 3 with other neurodegenerative diseases (1 normal pressure hydrocephalus, 2 cortico-basal syndrome)−according to the diagnostic criteria of each syndrome. The remaining subjects had negative CSF AD biomarkers and were diagnosed with stable mild cognitive impairment (*n* = 6), psychiatric diseases (*n* = 5) or subjective memory complaints (*n* = 5). Patients with a diagnosis of vascular dementia were excluded. The number of cardiovascular risk factors (diabetes, hypertension, hypercholesterolaemia, ischemic heart disease, previous transient ischemic attack/stroke, smoke) was rated in every included subject.

All patients underwent a brain MRI using a 3T unit (Philips Achieva, dStream, Eindhoven, Netherlands). Multiple MRI protocols were used, but all included three-dimensional T1-weighted images (matrix ≥ 192 × 192, slice thickness ≤ 1 mm) and three-dimensional fluid-attenuated inversion recovery (FLAIR) scan (matrix ≥ 224 × 224, slice thickness = 1 mm). Moreover, a lumbar puncture for the determination of Aβ_1–42_ (Aβ_42_), phosphorylated-tau at threonine 181 (pTau), total tau (tTau) and AQP4 levels in the CSF was performed in all subjects.

Enlarged perivascular spaces (EPVS) were visually rated by AA, a neurologist expert in neuroimaging and trained in EPVS counting, applying Standards for Reporting Vascular Changes on Neuroimaging (STRIVE) criteria (signal intensity similar to CSF on all sequences, adherence to the course of penetrating vessels, linear or round/ovoid, and a diameter smaller than 3 mm) (Wardlaw et al., 2013). According to Paradise et al. (2020), the number of EPVS in the slices 2 mm (BG) and 37 mm (CSO) above the anterior commissure were counted.

Lumbar puncture was performed in the L3/L4 or L4/L5 interspace between 8 and 10 am after a night of fasting. The samples were later centrifuged at 1,500 × *g* for 10 min at 4°C. The supernatants were aliquoted in polypropylene tubes and stored at –80°C until use. Before freezing them, the samples were analyzed and CSF cell count, glucose, and total protein levels were calculated. CSF levels of Aβ_42_, tTau, and pTau were assessed using either a ChemiLuminescence Enzyme ImmunoAssay (CLEIA) by a Lumipulse G600II platform (Fujirebio, Ghent, Belgium) or an ELISA method. Subjects for whom only older ELISA-based measurements were available were not included in subsequent analyses involving Aβ_42_ and tau proteins. AQP4 concentration in the CSF was determined with a specific ELISA kit from Cusabio for the whole population under study. The sensitivity of this assay or lower limit of detection of human AQP4 is typically less than 39 pg/ml, as reported by the manufacturer.^1^

Data were analyzed using the statistical spreadsheets Jamovi 1.6.23 and RStudio 2022.07.2. At visual inspection, the distribution of EPVS and all CSF biomarkers was positively skewed; hence, data were log-transformed for correlation and regression analyses. After transformation, distributions of EPVS numbers were inspected and outliers (defined as being more than 3 SDs from the mean) were excluded. Pearson’s correlation test was used, after checking for normality with Shapiro–Wilk test. Multivariate linear regression analysis was used to determine the independent effect of different CSF biomarkers [log_10_(x)-transformed] on EPVS number [log_10_(x + 1)-transformed], adjusting for demographic and clinical characteristics (age, sex, education, disease duration). Moreover, the confounding role of different MRI protocols and cardiovascular risk factors was controlled for. Results with a *p*-value of < 0.05 were considered statistically significant.

## Results

After excluding outliers (*n* = 3; 2 AD, 1 MCI) based on CSO-EPVS number, 67 subjects were included. Of them, 51 subjects had CLEIA-based measurements of Aβ_42_ and tau proteins available and were considered for analyses concerning AD biomarkers. Main demographic and clinical characteristics of the population under study are summarized in Table 1.

**TABLE 1:** Demographic and clinical data.

	Median	(Q1, Q3)
Age (years)	72	(65, 76)
Gender (F:M)	28:39	–
Education (years)	13	(8, 13)
Disease duration (years)	3	(2, 5)
Cardiovascular risk factors	1	(0, 2)
MMSE	25	(22, 27)
Total EPVS	44	(35, 62)
BG-EPVS	24	(17, 31.5)
CSO-EPVS	21	(13, 33)
AQP4 (pg/mL)	126.9	(98.9, 242.9)
Aβ_42_ (pg/mL)	576	(435, 824)
pTau (pg/mL)	63	(42, 138)
tTau (pg/mL)	447	(291, 845)

MMSE, mini mental state examination scores; EPVS, enlarged perivascular spaces; BG-EPVS, enlarged perivascular spaces in the basal ganglia; CSO-EPVS, enlarged perivascular spaces in the centrum semiovale; AQP4, aquaporin-4; Aβ42, amyloid-beta 1-42; pTau, phosphorylated-tau at threonine 181; tTau, total tau.

Aquaporin-4 (AQP4) levels were negatively associated with mini mental state examination scores at the time of lumbar puncture (*r* = −0.36, *p* = 0.003). No other significant associations between CSF biomarkers and clinic-demographic data were found.

**FIGURE 1:**
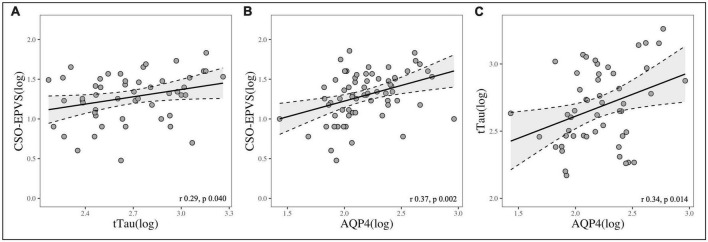
Scatterplots showing the correlation between: **(A)** CSO-EPVS number and CSF-tTau; **(B)** CSO-EPVS number and CSF-AQP4; **(C)** CSF-tTau and CSF-AQP4.

Total EPVS, BG-EPVS and CSO-EPVS burden showed a strong correlation with AQP4 levels (*r* = 0.38, *p* = 0.001; *r* = 0.27, *p* = 0.025; *r* = 0.37, *p* = 0.002, respectively), and CSO-EPVS were positively correlated with tTau (*r* = 0.29, *p* = 0.040). AQP4 and tTau were also positively correlated between each other (*r* = 0.34, *p* = 0.014) (Figure 1). Neither EPVS counts nor AQP4 levels were associated with Aβ_42_ nor pTau levels considering the whole group of study.

Multivariate linear regression analyses revealed that higher AQP4 (β = 0.37, standard error [SE] = 0.12, *p* = 0.003) and tTau (β = 0.30, SE = 0.15, *p* = 0.046) levels in the CSF were significantly and independently associated with the presence of CSO-EPVS. In addition, AQP4 was an independent predictor of greater BG-EPVS (β = 0.20, SE = 0.08, *p* = 0.009) and total EPVS (β = 0.28, SE = 0.08, *p* = 0.001) burden and of higher tTau levels in the CSF (β = 0.35, SE = 0.13, *p* = 0.008) (Table 2). AQP4 and tTau were still independent predictors of CSO-EPVS numbers after adjusting for different MRI protocols and cardiovascular risk factors (β = 0.28, SE = 0.13, *p* = 0.040; β = 0.35, SE = 0.15, *p* = 0.021, respectively).

**TABLE 2:** Linear regression models showing the predictive value of: **(A)** CSF-AQP4 on CSO-EPVS number and CSF-tTau levels; **(B)** CSF-tTau levels on CSO-EPVS number.

A.			AQP4	Age	Gender	Education	Dis. duration
	**CSO-EPVS**	β	0.37	0.01	0.05	−0.01	< 0.01
		SE	0.12	0.01	0.07	0.01	0.01
		*p*	0.003*	0.284	0.472	0.468	0.924
	**tTau**	β	0.35	0.01	−0.04	0.01	−0.01
		SE	0.13	0.01	0.08	0.01	0.01
		*p*	**0.008** *	0.108	0.563	0.397	0.173
**B.**			**tTau**	**Age**	**Gender**	**Education**	**Dis. duration**
	**CSO-EPVS**	β	0.30	< 0.01	0.06	−0.01	0.01
		SE	0.15	0.01	0.08	0.01	0.01
		*p*	**0.046** *	0.590	0.487	0.310	0.512

CSO-EPVS, enlarged perivascular spaces in the centrum semiovale; BG-EPVS, enlarged perivascular spaces in the basal ganglia; AQP4, aquaporin-4 in the CSF; tTau, total tau in the CSF; Dis. duration, disease duration. Bold values represent statistically significant *p*-values. *Indicate statistically significant *p*-values.

## Discussion

In this pilot study, we demonstrated that EPVS, particularly in the CSO, were strongly correlated with AQP4 levels in the CSF in a population of patients undergoing neurological work-up in suspicion of dementia. Moreover, we found that both CSO-EPVS counts and CSF-AQP4 levels were associated with levels of CSF-tTau, an established biomarker of neurodegeneration.

Given the close spatial and functional link between PVS and AQP4 according to the GS model (Iliff et al., 2012; Rasmussen et al., 2018), the hypothesis of a higher burden of EPVS being associated with increased AQP4 levels in the CSF seems highly plausible.

Several studies have described an association between MRI-visible PVS and neurodegenerative diseases, particularly in CSO white matter (Boespflug et al., 2018; Vilor-Tejedor et al., 2021; Perosa et al., 2022; Wang et al., 2022). Moreover, AQP4 is known to undergo over-expression and loss of perivascular localization in response to aging and AD pathologic changes (Zeppenfeld et al., 2017; Boespflug et al., 2018). In line with this, we previously described higher levels of CSF-AQP4 in AD and FTD patients compared to subjects not affected by neurodegenerative diseases (Arighi et al., 2022). This increase in AQP4 expression could represent either a compensatory mechanism by astrocytes to restore GS function or be secondary to reactive astrogliosis (Kress et al., 2014).

Regression analyses showed that higher levels of tTau in the CSF were predictive of a greater number of CSO-EPVS and were also associated with increased levels of CSF-AQP4.

Some previous works explored the association between EPVS and tau pathology, with conflicting results. In a study by Wang et al. (2021) a high degree of CSO-EPVS was associated with tau deposition in cognitively normal older people, as assessed by flortaucipir positron emission tomography (PET) positivity, but not Aβ deposition. Moreover, Vilor-Tejedor et al. (2021) found that CSO-EPVS were associated with higher levels of CSF pTau, tTau, and neurogranin in asymptomatic Aβ-positive participants. On the contrary, Kang et al. (2022) more recently described an inverse correlation between BG-EPVS and cerebral tau-PET signal and no relationship between CSO-EPVS and cerebral tau deposition in cognitively impaired Aβ-positive patients. In addition, Sepehrband et al. (2021) found an inverse association between PVS volume fraction and neurofibrillary tau deposition in the entorhinal cortex of cognitively impaired individuals.

Our data show a strong association between tTau and CSO-EPVS but not BG-EPVS. This finding seems biologically conceivable, since GS dysfunction may limit effective waste removal from the brain, including tau (Iliff et al., 2014), which can in turn clog up the system upstream, initiating a vicious cycle and leading to further reduction of perivascular drainage, PVS enlargement and ultimately neurodegeneration. The absence of correlation between tTau and BG-EPVS is also unsurprising and in keeping with previous literature data suggesting that BG-EPVS number may be more related to cardiovascular risk factors and aging rather than neurodegeneration (Bown et al., 2022; Evans et al., 2023).

Regarding the relationship between tau and AQP4, our group already described a positive correlation between the CSF levels of tTau and AQP4 (Arighi et al., 2022). Since populations analyzed are non-overlapping, findings from the present study can be considered confirmatory.

Although our research is rather preliminary and needs replication, our findings provide evidence that MRI-visible CSO-EPVS and AQP4 might be clinically meaningful biomarkers of glymphatic dysfunction and associated neurodegeneration. In fact, though the exact pathophysiology of PVS enlargement is still poorly understood, our data may suggest that a failure to eliminate waste products and abnormal proteins through the glymphatic pathway may lead to PVS dilation on one hand and hasten neurodegeneration on the other.

Limitations of this study include the small sample size and its cross-sectional design, which prevents conclusions on the temporal and causal sequence of the events observed. In addition, different protocols were used for MRI acquisition, as a direct consequence of the retrospective nature of the study and its clinical setting. However, the confounding effect of different protocols was controlled for in regression models. A third limitation is that, although several PVS rating methods have been described, we used a visual rating scale applied by a single rater and restricted the quantification of PVS to two brain regions, BG and CSO. Though automated quantitative techniques for EPVS quantification exist (Ballerini et al., 2018; Sepehrband et al., 2019) and have been shown to have better sensitivity (Barisano et al., 2022), Paradise et al.’s (2020) visual rating scale and STRIVE criteria have been thoroughly validated. Another limitation is the lack of a standardized methodology for the measurement of AQP4. Given both the presence of some data arguing against the detectability of AQP4 in the CSF (Hiraldo-González et al., 2021) and the absence of confirmatory data from other groups, we cannot draw definitive conclusions on the inter-laboratory reproducibility of AQP4 dosage. However, the consistency of the results between our two studies, which analyzed two completely different cohorts of patients, provides reassuring evidence of reliability.

Our study has several strengths as well: it is one of the first exploring the correlation between putative biomarkers of the newly described GS; it focuses on easily accessible biomarkers since both MRI and lumbar puncture are already widely used in the diagnostic workup of people with suspected dementia; data were highly homogeneous since all CSF analyses were performed in the same laboratory and all MRI images were acquired with standardized protocols in a single MRI scanner.

## Data availability statement

The raw data supporting the conclusions of this article will be made available by the authors, without undue reservation.

## Ethics statement

The studies involving human participants were reviewed and approved by the Comitato Etico Area 2 Milano, approval N 859_2021, date 14.09.2021. The patients/participants provided their written informed consent to participate in this study.

## Author contributions

LS and AA designed the study, analyzed, and interpreted the data. LS drafted the manuscript. TC, MP, GF, and AP contributed to the analysis and interpretation of the data. MA, CF, MS, FS, and CV performed CSF analyses. GC and VC helped in the analysis of MRI data. FT acquired MRI data. ES and DG drafted and revised the manuscript for intellectual content. All authors read and approved the final manuscript.

## Footnotes

1
www.cusabio.com
